# Non-perfusing cardiac rhythms in asphyxiated newborn piglets

**DOI:** 10.1371/journal.pone.0214506

**Published:** 2019-04-04

**Authors:** Anne Lee Solevåg, Deandra Luong, Tze-Fun Lee, Megan O’Reilly, Po-Yin Cheung, Georg M. Schmölzer

**Affiliations:** 1 Department of Paediatric and Adolescent Medicine, Akershus University Hospital, Lørenskog, Norway; 2 Centre for the Studies of Asphyxia and Resuscitation, Neonatal Research Unit, Royal Alexandra Hospital, Edmonton, Alberta, Canada; 3 Faculty of Science, University of Alberta, Edmonton, Alberta, Canada; 4 Department of Pediatrics, Faculty of Medicine and Dentistry, University of Alberta, Edmonton, Alberta, Canada; Western College of Veterinary Medicine, University of Saskatchewan, CANADA

## Abstract

**Aim:**

We recently demonstrated that asphyxiated piglets commonly had bradycardia displayed on electrocardiography (ECG) while no carotid blood flow (CBF) or audible heart sounds could be detected. Such pulseless electrical activity (PEA) in newborn infants has not previously been thoroughly described. The aim of this study was to further investigate the occurrence of non-perfusing cardiac rhythms in asphyxiated piglets and the potential implications for the success of cardiopulmonary resuscitation (CPR) and short-term survival.

**Methods:**

Neonatal piglets (1–4 days, 1.7–2.4kg) had their right common carotid artery exposed and enclosed with a real-time ultrasonic flow probe. Heart rate (HR) was continuously measured and recorded using ECG. This allowed simultaneous monitoring of HR via ECG and CBF. The piglets were asphyxiated until cardiac arrest, defined as no CBF and no audible beat upon precordial auscultation. CPR was performed until return of spontaneous circulation (ROSC, defined as a HR ≥100 bpm). ECG traces were retrospectively assessed.

**Results:**

Nine out of 21 piglets (43%) had QRS-complexes on their ECG while no CBF and no audible heart sounds could be detected. Five (56%) of the piglets with PEA and 12/12 (100%) piglets with asystole at cardiac arrest obtained ROSC (p = 0.02). Thirty-three per cent of the piglets with PEA versus 58% with asystole survived to 4 hours post-ROSC (p = 0.39).

**Conclusion:**

Cardiac arrest in the presence of a non-perfusing cardiac rhythm on ECG is common in asphyxiated piglets. Clinical arrest in the presence of a non-perfusing cardiac rhythm on ECG may reduce the success of CPR.

## Introduction

Adult out-of-hospital cardiac arrests (OHCA) are commonly of primary cardiac origin with ventricular fibrillation (VF) as the cause of arrest. Thus, rhythm diagnosis and defibrillation are important features of adult cardiopulmonary resuscitation (CPR) [1]. In contrast, paediatric cardiac arrest is usually of respiratory aetiology, and the initial rhythm is often non-shockable including asystole and pulseless electrical activity (PEA) [2]. Therefore, the initial focus for paediatric OHCA has been on rescue breathing and chest compressions whereas rhythm diagnosis and defibrillation have received less emphasis. However, in observational studies, VF was diagnosed as the initial rhythm in 4–19% of paediatric cardiac arrests [3, 4].

When direct evidence is lacking, guidelines for paediatric CPR are developed with consideration of the evidence from adults [5]. In the neonatal subpopulation, even less direct evidence exists, and guidelines for neonatal resuscitation are rather simplified compared to adult guidelines; e.g., adrenaline (epinephrine) is the only drug in the neonatal resuscitation algorithm [6, 7]. Antiarrhythmic medications, such as amiodarone and lidocaine, or defibrillation are not considered during neonatal CPR; mainly because shockable arrhythmias such as VF and pulseless ventricular tachycardia (pVT) have not been recognized in newborn infants with cardiac arrest.

Pulseless electrical activity (PEA) is organized cardiac electrical activity without associated mechanical activity [8]. Treatment includes reversing the cause of cardiac arrest [9], in addition to providing assisted ventilation and chest compression. The PEA rhythm may be sinus, atrial, junctional, or ventricular in origin, but is broadly categorized as narrow QRS-complex (70% of cases) and wide complex PEA [9]. Narrow complex PEA on electrocardiography (ECG) may be caused by a mechanical problem due to right ventricle inflow or outflow obstruction (e.g., cardiac tamponade, tension pneumothorax, mechanical lung hyperinflation, and pulmonary embolism), whereas wide complex PEA is more likely to be due to a metabolic condition (e.g., hyperkalaemia and sodium channel blocker overdose), left ventricular failure (due to ischemia), or an agonal rhythm (clinically regarded as asystole with equivalent treatment approach) [10]. PEA may also be caused by hypovolaemia, tachydysrhythmias, and cardiomyopathy [8]. It is stated that only a very small percentage of PEA arrests are caused by asphyxia [11]. However, we recently demonstrated that in severely asphyxiated piglets, 23/54 (43%) of the animals had distinct QRS-complexes on the ECG without a detectable carotid blood flow or an audible heartbeat on precordial auscultation [12, 13]. In addition, recent case reports [14, 15] reported five cases of PEA in newborn infants during neonatal resuscitation in the delivery room. Most concerning, 4/5 infants died during resuscitation. The aim of the present study was to further examine the occurrence of PEA and potentially other arrhythmias in asphyxiated piglets. Based on the poor outcome after PEA in adults [16] and older children [5], we hypothesized that PEA negatively influences the success of CPR and short-term survival of asphyxiated piglets.

## Materials and methods

Secondary analysis of a previously published randomized animal trial in asphyxiated piglets using different methods of CPR [17].

### Subjects

Newborn mixed breed piglets (1–4 days, 1.7–2.4 kg, n = 41) were obtained on the day of experimentation from the Swine Research Technology Center, University of Alberta. All experiments were conducted by certified University of Alberta Animal User Training Program researchers, and conducted in accordance with the guidelines. The research was approved by the Animal Care and Use Committee (Health Sciences), University of Alberta and presented according to the ARRIVE guidelines [18]. The protocol is presented in [17].

### Animal preparation

The piglets were anesthetized with Isoflurane 1–5%, tracheotomised and mechanically ventilated (Sechrist infant ventilator model IV-100; Sechrist Industries, Anaheim, CA) at a 25/min rate, peak inspiratory pressure of 25cmH_2_O and positive end-expiratory pressure of 5cmH_2_O. After central vascular access was obtained, hydration was maintained with 5% Dextrose and 0.9% NaCl, and anaesthesia was changed to intravenous morphine 50-200mcg/kg/h and propofol 0.1–0.2mg/kg/h. A bolus of morphine (0.15mg/kg) was given before tracheotomy. Piglets recovered from surgical instrumentation for 1h during which the ventilator rate and airway pressure were adjusted to keep paCO_2_ 35–45mmHg.

### Surgical procedures

A 5-French Argyle single-lumen catheter (Covidien, Dublin, Ireland) was inserted into the left common carotid artery (CCA) for continuous blood pressure monitoring and blood sampling. A 5-French Argyle double-lumen catheter (Covidien) was inserted in the external jugular vein on the same side for fluid and medication infusion. The piglet was tracheotomised and a 3.5 uncuffed endotracheal tube was inserted and fixed to the trachea. A real-time ultrasonic flow probe (2SB; Transonic Systems Inc., Ithaca, NY) was placed around the right CCA. Systemic arterial pressure and heart rate (HR) were measured continuously with a Hewlett Packard 78833B monitor (Hewlett Packard Co., Palo Alto, CA).

### Experimental protocol

Asphyxia was induced as described in [17] by reducing FiO_2_ to 0.08 and reducing the ventilator rate by 10/min every 10min until a rate of 0/min was reached. Ten minutes later, the ventilator was disconnected and the endotracheal tube clamped until cardiac arrest/asystole, defined as carotid blood flow <5 mL/min and no audible HR upon auscultation of the precordium [17].

Thirty seconds after cardiac arrest was diagnosed, we provided positive pressure ventilation (PPV) with air for 30sec with a Neopuff T-Piece (Fisher & Paykel, Auckland, NZ) with peak inspiratory pressure 25cmH_2_O and positive end-expiratory pressure 5cmH_2_O before chest compression (CC) was started. Manual CC was performed and PPV provided at a 30/min rate. If there was no return of spontaneous circulation (ROSC) after 30sec of CC, adrenaline (0.02mg/kg) was given intravenously and repeated every 3min as needed (maximum 4 doses). CPR was discontinued if ROSC was not achieved after 15min. As previously described [19], ROSC was defined as an unassisted HR ≥ 100 bpm demonstrated by arterial blood pressure waveforms. After ROSC, piglets were observed for 4h and euthanized (within five minutes) with IV phenobarbital (100 mg/kg), unless death occurred earlier. Humane endpoints included a decrease in HR <100 bpm or hypotension, and decrease in haemoglobin <5.5 g/dL. No animal died before meeting criteria for euthanasia.

### Data collection and analysis

We recorded age, weight and sex of the piglets. Transonic flow probe, HR and pressure transducer outputs were digitized and recorded (PowerLab LabChart software (ADInstruments, Dunedin, NZ)). Cardiac output was measured with echocardiography (Vivid 7/5S probe (GE Healthcare, Buckinghamshire, UK)) at baseline, during asphyxiation, and 30 min and 4 h after ROSC as described in [17]. Markers for cardiac arrest were placed within the LabChart program to indicate the time of cardiac arrest before initiation of the resuscitation protocol. This marker was then used to compare timing of onset of arrest as determined by auscultation, ECG and CBF. The study is based on secondary analyses of a ROSC study in asphyxiated piglets [17]. Continuous variables are presented as median (interquartile range (IQR)). Data was compared between groups using the Mann-Whitney *U* test for continuous variables, and χ^2^ for categorical variables. *P*-values were 2-sided and p<0.05 was considered statistically significant. Statistical analyses were performed with IBM SPSS 25 for Mac (IBM Corporation, Armonk, NY).

## Results

**Fig 1:**
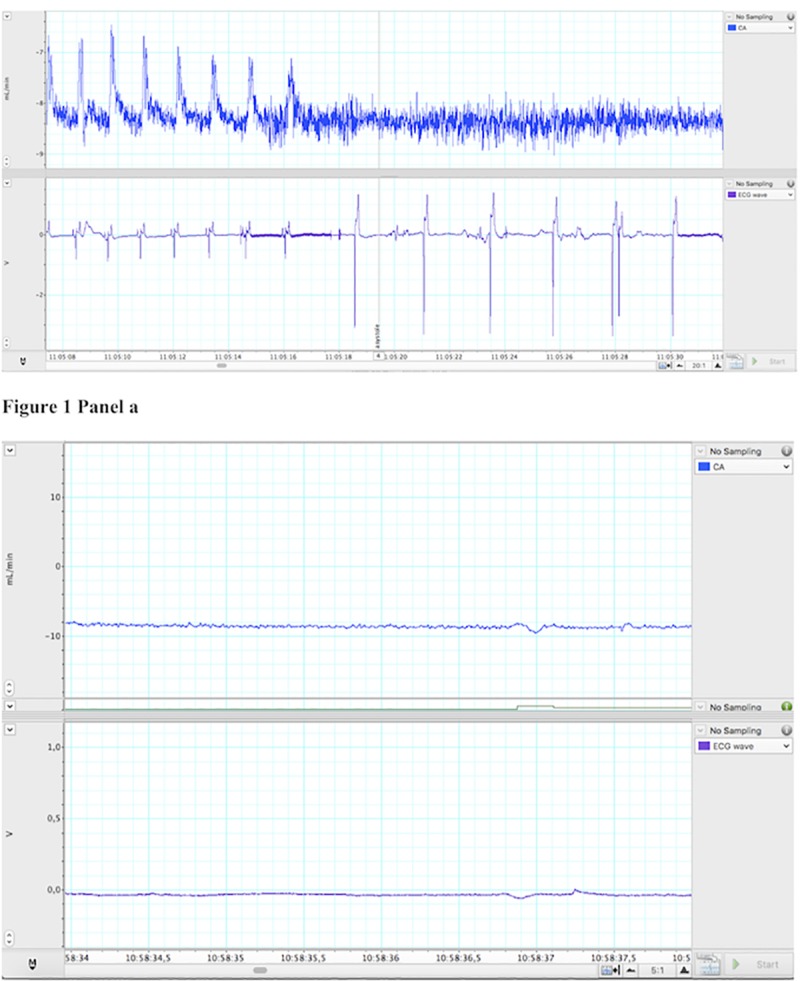
Waveforms of carotid artery (CA) blood flow (CBF) and electrocardiogram (ECG). Panel a: ECG showing bradycardia in the absence of CBF and no audible sound. Panel b: Asystole correctly assessed with absence of CBF, ECG and no audible heart sound.

Thirty-two piglets were analysed with respect to ECG rhythm at the time of cardiac arrest. At cardiac arrest, median (IQR) arterial pH was 6.6 (6.6–6.7), paCO_2_ was 91 (54–101) mmHg, base excess -28 (-30-(-25)) mmol/L, and lactate was 18 (17–20) mmol/L. In 11 (34%) piglets, the ECG tracings were of insufficient quality for an interpretation to be made. In the piglets where ECG failed to give a reliable signal at cardiac arrest, the duration of hypoxia/asphyxia had been longer than in the piglets with ECG tracings of good quality (42 (32–43) vs 33 (26–34) min, p = 0.007). However, pH at cardiac arrest (p = 1.00), and time to ROSC (p = 0.89) were not different between piglets with insufficient vs. good quality ECG tracings. Piglets with good quality ECG tracings survived the whole experiment in 10/21 (48%) of cases, vs. 2/11 (18%) piglets with insufficient quality ECG (p = 0.14). Of the 21 piglets with good quality ECG, nine (43%) had identifiable QRS-complexes on their ECG while no CBF and no audible heart rate could be detected (Fig 1A). The QRS-rate ranged from 38 to 190 beats per minute (median: 66 beats per minute). In all cases, the QRS-complexes were interpreted as narrow-complex PEA. None of the piglets had VF or pVT. Twelve (57%) piglets were asystolic with no QRS-complexes visible on the ECG at the time of arrest (Fig 1B).

**Fig 2:**
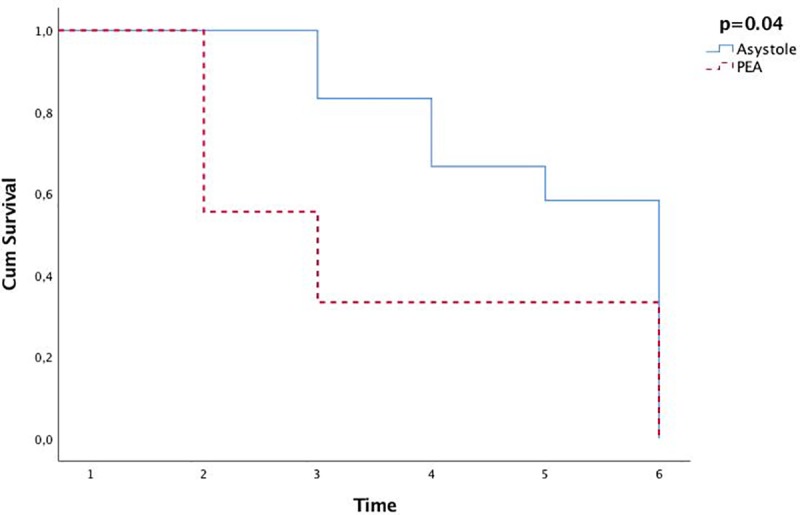
Kaplan–Meier survival graph for piglets with pulseless electrical activity (PEA) and asystole p = 0.04. 1 = start of experiment, 2 = CPR, 3 = 1h after return of spontaneous circulation (ROSC), 4 = 2h after ROSC, 5 = 3h after ROSC, 6 = 4h after ROSC.

Characteristics of the piglets with good quality ECG-recordings are presented in Table 1. pH (6.5 (6.5–6.8) vs. 6.6 (6.6–6.7), p = 0.42) and lactate (18 (14–20) mmol/L vs. 18 (17–20) mmol/L, p = 0.88) at the time of cardiac arrest were similar in piglets with PEA and asystole, respectively. There was no difference in the distribution of CPR interventions (original study of different oxygen fractions and CC methods) between piglets with PEA and asystole. Time to ROSC was not different between piglets with PEA and asystole, but the fraction of piglets obtaining ROSC was lower in piglets with PEA compared to asystole (Table 1). Three out of nine (33%) piglets with PEA survived to 4 hours post-ROSC, whereas seven out of 12 (58%) piglets with asystole survived (Table 1). There was no difference in HR (Table 1) and MAP (Table 2) at 4 hours post-ROSC between piglets with PEA and asystole (Table 1). Fig 2 is a Kaplan-Meyer survival graph showing that piglets with PEA died earlier during the course of the experiment than the piglets with asystole (p = 0.04).

**Table 1:** Characteristics of piglets with pulseless electrical activity (PEA) versus asystole on electrocardiogram at cardiac arrest.

	PEA (n = 9)	Asystole (n = 12)	p-value
Sex (female/male)	3/6	7/5	0.39
Age (days)	2 (2–3)	2 (1–3)	1.00
Weight (kg)	1,9 (1,8–2,3)	2,0 (1,7–2,3)	1.00
Baseline HR (bpm)	230 (202–268)	198 (179–238)	0.31
Hypoxia/asphyxia time (min)	33 (31–37)	31 (26–33)	0.68
ROSC (Y/N)	5/4	12/0	0.02
Time to ROSC (sec)	170 (92–182)	117 (95–25)	0.92
Adrenaline doses (n)	2 (0.5–4)	1 (0–1)	0.22
Survival to 4 hours (n (%))	3 (33)	7 (58)	0.39
HR at 4 hours (bpm)	249 (196-)*	229 (219–234)	0.55

Continuous variables are reported as median (interquartile range)

HR–heart rate

ROSC–return of spontaneous circulation

*not able to calculate interquartile range (n = 3)

**Table 2:** Hemodynamic variables in piglets with pulseless electrical activity (PEA) versus asystole reported as median (interquartile range).

	Baseline		20 min asphyxia		30 min after ROSC		4 h after ROSC	
	PEA	Asystole	PEA	Asystole	PEA	Asystole	PEA	Asystole
CA flow (mL/min)	86 (72–104)	76 (61–94)	78 (56–93)	68 (36–81)	31 (22-)*	39 (23–50)	12 (0-)*	16 (3–23)
MAP (mmHg)	80 (76–90)	76 (69–85)	70 (45–76)	55 (48–64)	70 (67-)*	55 (53–63)	32 (31-)*	43 (24–54)
CVR (mmHg·mL·min^-1^)	0,97 (0,93-)*	1,07 (0,90–1,32)	0,82 (0,79-)*	0,79 (0,65–1,19)	2,17 (1,16-)*	1,64 (1,06–2,35)	2,69 (2,58-)*	2,78 (1,65–9,75)
CO (mL/kg/min)	309 (257–450)	341 (237–345)	196 (156–581)	216 (95–299)	240 (227-)*	216 (188-)*	66 (7-)*	134 (111-)*

The differences between asystole and PEA were not significant for all variables at all time points.

CA–carotid artery

MAP–mean arterial blood pressure

CVR–carotid artery vascular resistance

CO–cardiac output

ROSC–return of spontaneous circulation

*not able to calculate interquartile range (n = 3)

## Discussion

In this study of asphyxia-induced cardiac arrest, we observed that piglets frequently had detectable QRS-complexes on ECG while there were no CBF and audible heart contractions (auscultation). Our findings are similar to previous reports in asphyxiated piglets with 40–50% having PEA after asphyxia-induced cardiac arrest [12, 13].

Initial non-shockable rhythms (PEA or asystole) account for about two-thirds of adult OHCA with an increasing incidence [11] compared to initial shockable rhythms (VF and pVT) [20, 21]. Overall survival after adult OHCA is about 8% [22], with a worse prognosis with PEA compared to initial shockable rhythms [16, 23–28]. Even if the rhythm converts from non-shockable to shockable during CPR, outcomes (e.g., survival to hospital discharge) do not improve [21, 29]. Similarly, during paediatric cardiac arrest, a shockable rhythm (VT/pVT) is a predictor for improved outcome [5]. We previously reported that 1/54 asphyxiated piglets had VT/VF with no CBF or audible heart sounds [12]. In the present study, no piglet had a shockable rhythm. In all the piglets with PEA, we only observed narrow QRS-complexes on the ECG. We speculate that asphyxia, and potentially hypovolaemia, are associated with narrow-complex PEA. Similar to human adults and older children, PEA resulted in less asphyxiated piglets achieving ROSC and survival compared to asystole.

A chart review of 262 adults with cardiac arrest and an initial rhythm of PEA reported that neither electrical rate nor QRS width was associated with survival or neurologic outcome [9]. However, there was a trend toward improved survival in bradycardic PEA compared to other PEA rhythms (i.e., normocardic or tachycardic PEA). Unorganized PEA may represent a final common preterminal electrical rhythm. PEA in our piglets had an electric QRS heart rate ranging between 38 to 190 per minute. However, the sample was too small to be stratified to bradycardic versus normocardic versus tachycardic PEA.

Our study is hypothesis generating how the initial ECG-rhythm might affect the prognosis of asphyxiated newborn infants that require delivery room CPR. Questions that remain unanswered include i) whether there is a difference in disease severity between infants with asystole versus PEA, or ii) whether PEA in itself affects the myocardial response to resuscitative measures. The piglets with PEA had the same hypoxia time and similar biochemical signs of asphyxia compared to piglets with asystole. However, piglets with PEA had a poorer response to CPR with only about half the piglets obtaining ROSC.

Newborn piglets have similar anatomy and pathophysiology to newborn infants at near-term gestation. In addition to anaesthetic and surgical confounding factors, all piglets had already undergone foetal to neonatal transition, and their responses to severe asphyxia may not be entirely comparable to infants during foetal-to-neonatal transition. A perivascular flow probe was placed around the right CCA while the left CCA was cannulated for MAP measurements and blood sampling. Although this approach has been used in previous animal models of perinatal asphyxia [30], occluding the left CCA could potentially change the flow through the right CCA, resulting in abnormal flow values relative to a non-occluded state. We still argue that the lack of difference in CCA flow between asystolic and PEA piglets may be valid.

The difference in survival to 4 hours after ROSC between the PEA and asystole groups did not reach statistical significance, which was potentially due to a small sample size. As our results are based on secondary analyses of a study with a different endpoint, a power calculation was not performed for 4-hour survival.

### Clinical applicability

ECG was only recently introduced to the delivery room [6]. Recent guidelines have suggested the potential benefit of ECG monitoring as standard of care due to the faster acquisition of a HR signal in preterm infants [31], and better accuracy compared to pulse oximetry [32]. However, the clinical data was collected mainly in non-asphyxiated infants.

Our findings in piglets indicate that in one-third of the cases, ECG fails to provide a signal when the asphyxia becomes severe. In piglets with good quality ECG recordings, ECG demonstrated a non-perfusing rhythm, so-called PEA, in more than a third of cases. During perinatal asphyxia, any ECG HR without simultaneously assessing clinical signs of perfusion using auscultation or palpation should be considered suspicious of PEA. Based on the high incidence of a non-perfusing rhythm observed in our asphyxiated piglets, ECG rates alone might not be optimal to guide CPR interventions in asphyxiated infants. For adult use, efforts are made to develop devices and methods that may facilitate rhythm interpretation and decrease hands off time during CC [33]. In newborn infants, novel methods for HR assessment include digital stethoscopes or Doppler ultrasound [34–37]. Both technologies can obtain a HR faster than pulse oximetry [35–37] and have a good correlation with ECG HR [35, 37]. Bowel gas or movement of the infant might interfere with the signal acquisition using Doppler [36], while crying can decrease the accuracy of digital stethoscope [37]. However, neither movements nor crying are present in unresponsive newborn infants who require resuscitation. While both technologies have been assessed in healthy term and preterm infants, neither was assessed in asphyxiated infants. Further studies are needed before they could be introduced into clinical care.

HR assessment remains central to neonatal CPR. However, failure to recognize the difference between PEA and bradycardia on an ECG when assessing HR without using other signs of systemic perfusion might be detrimental. The focus of neonatal CPR should remain on ventilation and chest compressions, as shockable rhythms are very rare in this population.

## Conclusion

Cardiac arrest in the presence of a non-perfusing cardiac rhythm (PEA) on ECG was common in asphyxiated piglets. Piglets with PEA had lower rates of ROSC and lower 4 h survival compared to asystole, but this did not reach statistical significance.

We recommend against the use of ECG as the sole method for assessing HR in the delivery room. Our study indicates that a combination of techniques or methods should be used to assess perfusion during neonatal resuscitation. Our results should guide future efforts to investigate heart rhythm disturbances and arrhythmias in newborn infants in the delivery room.

## Supporting information

S1 Dataset The dataset generated for this study. (SAV)

## Abbreviations

OHCA: Out-of-hospital cardiac arrests
VF: Ventricular fibrillation
CPR: Cardiopulmonary resuscitation
PEA: Pulseless electrical activity
pVT: Pulseless ventricular tachycardia (pVT)
ECG: Electrocardiography
CA: Carotid artery
HR: Heart rate
CBF: Carotid blood flow
PPV: Positive pressure ventilation
CC: Chest compression
ROSC: Return of spontaneous circulation
